# Pyrosequencing for rapid detection of Tuberculosis resistance in clinical isolates and Sputum samples from re-treatment Pulmonary Tuberculosis patients

**DOI:** 10.1186/1471-2334-14-200

**Published:** 2014-04-13

**Authors:** Ruijuan Zheng, Changtai Zhu, Qi Guo, Lianhua Qin, Jie Wang, Junmei Lu, Haiyan Cui, Zhenling Cui, Baoxue Ge, Jinming Liu, Zhongyi Hu

**Affiliations:** 1 Shanghai Key Laboratory of Tuberculosis, Shanghai Pulmonary Hospital, Tongji University School of Medicine, No. 507 Zhengmin Rd, Shanghai 200433, People's Republic of China; 2 Department of Respiratory Medicine, Shanghai Pulmonary Hospital, Tongji University School of Medicine, No. 507 Zhengmin Rd, Shanghai 200433, People’s Republic of China; 3 Department of Laboratory Medicine, Shanghai Jiao Tong University Affiliated Sixth People’s Hospital, No. 600 Yishan Rd, Shanghai 200233, China

## Abstract

**Background:**

Multidrug-resistant tuberculosis (MDR-TB) is a major public health problem. Early diagnosis of MDR-TB patients is essential for minimizing the risk of *Mycobacterium tuberculosis* (MTB) transmission. The conventional drug susceptibility testing (DST) methods for detection of drug-resistant *M.tuberculosis* are laborious and cannot provide the rapid detection for clinical practice.

**Methods:**

The aim of this study was to develop a pyrosequencing approach for the simultaneous detection of resistance to rifampin (RIF), isoniazid (INH), ethambutol (EMB), streptomycin (SM), ofloxacin (OFL) and amikacin (AMK) in *M. tuberculosis* clinical isolates and sputum samples from re-treatment pulmonary tuberculosis (PTB) patients. We identified the optimum conditions for detection mutation of *rpoB*, *katG*, *rpsl*, *embB*, *gyrA* and *rrs* gene by pyrosequencing. Then this approach was applied to detect 205 clinical isolates and 24 sputum samples of *M. tuberculosis* from re-treatment PTB patients.

**Results:**

The mutations of *rpoB* and *gyrA* gene were detected by pyrosequencig with the SQA mode, and the mutations of *katG*, *rpsl*, *embB*, *gyrA* and *rrs* gene were detected by pyrosequencing with SNP mode**.** Compared with the Bactec MGIT 960 mycobacterial detection system, the accuracy of pyrosequencing for the detection of RIF, INH, EMB, SM, AMK and OFL resistance in clinical isolates was 95.0%, 79.2%, 70.3%, 84.5%, 96.5% and 91.1%, respectively. In sputum samples the accuracy was 83.3%, 83.3%, 60.9%, 83.3%, 87.5% and 91.7%, respectively.

**Conclusions:**

The newly established pyrosequencing assay is a rapid and high-throughput method for the detection of resistance to RIF, INH, SM, EMB, OFL and AMK in *M.tuberculosis*. Pyrosequencing can be used as a practical molecular diagnostic tool for screening and predicting the resistance of re-treatment pulmonary tuberculosis patients.

## Background

The emergence and spread of MDR-TB and extensively drug-resistant tuberculosis (XDR-TB) posed a severe impediment for the prevention and control of tuberculosis (TB) transmission [1-3]. According to the World Health Organization (WHO) [4], there were about 650,000 cases of MDR-TB present in the world in 2010, and was estimated that about 9% of these cases of XDR-TB. Annually, about 440,000 were infected with MDR-TB and 150,000 died of MDR-TB. Due to the severity of MDR-TB, the WHO convened a international meeting focused on MDR-TB in April 2009 in Beijing, China, urging member states to take detailed and effective action in diagnosing and treating M/XDR-TB [4]. Another survey of WHO in 2010 showed that the estimated MDR-TB among the notified re-treatment cases (n = 7,795) was 2,200 (28%) [5]. So the Global Plan to STOP TB (2006–2015) proposed that by 2015 all countries should carry out DST for all re-treatment tuberculosis patients [6]. In order to control the MDR-TB,it was important for re-treatment PTB patients to develop a rapid, reliable and high-throughput drug susceptibility testing to obtain first- and second-line anti-tuberculosis drugs susceptibility result simultaneously, as it was essential to decrease patient morbidity and mortality as well as treatment-associated costs.

At present, the conventional drug susceptibility testing methods for *M.tuberculosis* were the bacteriological methods performed by culture in liquid or on solid media with highly sensitive and specific to most anti-TB drugs. However, these methods were laborious, time-consuming and could not provide rapid detection for clinical practice. Consequently, the rapid molecular diagnostic methods which performed within 1 or 2 days would be helpful for shortening the detection time.

Spontaneous chromosomal mutations were the genetic basis for drug resistance in *M. tuberculosis*[7,8]. At present, RIF resistance was almost entirely associated with the mutation of an 81-bp region of the *rpoB* gene, the RIF resistance determining region (RRDR) [9-11]. The resistance to INH, EMB, SM, OFL and AMK was primarily found in mutations in the *katG*, *embB, rpsl, gyrA* and *rrs* genes, respectively [8,12-16]. All these resistance-associated mutations were predominantly found in a short region or a position in *M. tuberculosis* genome and could be considered as the beacons of molecular drug susceptibility testing. Currently, the WHO endorsed some molecular methods to detect individual anti-TB drugs susceptibility by detecting gene mutations associated with drug resistance, such as Xpert MTB/RIF [17] and GenoType MTBDR [18] methods.

Pyrosequencing was a DNA sequencing technique that was based on the detection of the pyrophosphate released during DNA synthesis [19-21], and was well suitable for large-scale screening for a short length DNA fragment. At present, pyrosequencing had become a novel approach for the detection of TB infection and tuberculosis resistance [22-25]. Compared to GenoType MTBDR method, pyrosequencing was more accurate and easier to handle. Compared to Xpert MTB/RIF method, pyrosequencing could detect more drugs resistance. In this study, our aim was to establish a rapid molecular method for the detection of tuberculosis resistance by pyrosequencing technology and to evaluate its clinical value in re-treatment pulmonary tuberculosis patients.

## Methods

### Ethics considerations

The Shanghai Pulmonary Hospital Affiliated to Tongji University School of Medicine Ethics Committee approved the research protocols (permit number: 2010–034). The informed consent was obtained from each patient who was treated in accordance with the Helsinki Declaration on the participation of human subjects in medical research.

### Source of clinical isolates and sputum samples

A total of 205 clinical *M.tuberculosis* isolates and 24 sputum samples were obtained from PTB patients in Shanghai Pulmonary Hospital from January 2010 to March 2011. All the sputum specimens were smear-positive and were classified according to an acid-fast bacillus (AFB) staining scale as 1+ (1 case), 2+ (3 cases), 3+ (12 cases) and 4+ (8 cases). All of the samples were identified as *M.tuberculosis* by Bactec MGIT 960 culture, Ziehl-Neelsen staining and standard biochemical identification testing [26] and all of the samples were collected from re-treatment PTB patients. The re-treatment PTB definition was a patient who failed the standard initial tuberculosis treatment regimen, who relapsed after initial treatment with a standard or non-standard tuberculosis regimen. In addition, 10 clinical isolates, including 3 isolates susceptibility and 2 isolates resistant to RIF, INH, EMB, SM, AMK and OFL and the other 5 isolates resistant to four or five drugs, were collected from Shanghai Pulmonary Hospital. These isolates were sequenced on an ABI 3730xl DNA Analyzer by the Sangon Engineering Company (Shanghai, China) to obtain the sequence of *rpoB*, *katG, embB, rrs, gyrA* and *rpsl* genes. *M.tuberculois* standard strain (H37Rv, ATCC25618) was purchased from the National Culture Collection.

### Specimen processing and genomic DNA extraction

For *M.tuberculosis* isolates, genomic DNA extraction was performed as described previously [22]. For sputum samples, prior to DNA extraction, each sputum sample was decontaminated with an equal volume of 2% NaOH and 0.5% NALC, incubated at room temperature for 20 min, neutralized with 67 mM phosphate buffer (pH 6.8) and then centrifuged at 3,300 × *g* for 20 min at 4°C. The processed sediment was washed once using a sterile 0.9% NaCl solution and re-suspended in 1.0 ml sterile 0.9% NaCl solution. Two separate 500 μl aliquots were prepared in 1.5 ml tubes for the DNA extraction and Bactec MGIT 960 culture. The 500 μl aliquots for the DNA extraction were inactivated and lysed for 30 min at 85°C and DNA extraction was performed as described above for the extraction of clinical isolates previously. The concentration of the genomic DNA was measured by the Biophotometer (Eppendorf).

### Design of primers and PCR amplification

Regions of the *rpoB*, *katG, embB, rrs, gyrA* and *rpsl* genes were amplified by conventional PCR. Amplification and sequencing primers for pyrosequencing were designed with PSQ assay design software (Biotage, Inc., Charlottesville, VA). Primers and amplicon sizes were presented in Table 1. Amplification reaction was performed in a final volume of 50 μl consisting of 0.5 μl 10 × PCR buffer, 0.2 mM deoxynucleotide triphosphates (Takara), 1U Taq DNA polymerase (Takara), 200 nM of each primer, and 10 ng of DNA. Thermo-cycling conditions were as follows: 95°C for 3 min followed by 50 cycles of 30 s at 95°C, 30 s at respective annealing temperature, 30 s at 72°C and final elongation at 72°C for 10 min.

**Table 1:** Primers and thermo-cycling conditions of the pyrosequencing assays

**Drug**	**Gene**	**Primer**	**Sequences (5’—3’)**	**Annealing temperature (°C)**	**Size (bp)**	**Target loci**
RIF	*rpoB*	Forward	GTCCGGGAGCGGATGACCACCC	65	204	RRDR
		Reverse*	GCTCACGTGACAGACCGCCG			
		Sequencing 1	GCGATCAAGGAGTTC			
		Sequencing 2	TCATGGACCAGAACAA			
INH	*katG*	Forward	AGATGGGCTTGGGCTGGA	61	133	315
		Reverse*	TAGCCGTACAGGATCTCGAGGA			
		Sequencing	CCGGTAAGGACGCGA			
SM	*rpsl*	Forward*	CAAGGGTCGTCGGGACAAGA	61	299	
		Reverse	TCTTGACACCCTGCGTATCC			
		Sequencing 1	CGCCGAGTTCGGCTT			43
		Sequencing 2	CACACCAGGCAGGTC			88
EMB	*embB*	Forward	ACGACGGCTACATCCTGG	61	110	306
		Reverse*	GTTGTAATACCAGCCGAAGGGA			
		Sequencing	CGACGGCTACATCCTG			
OFL	*gyrA*	Forward	TTCGATTCCGGCTTCCGCCC	61	192	QRDR
		Reverse*	TGGGTCATTGCCTGGCGAGC			
		Sequencing	TACCACCCGCACGGC			
AMK	*rrs*	Forward	TCCTTAAAAGCCGGTCTCAGTTC	61	239	1401
		Reverse*	TCCGGTACGGCTACCTTGTTA			
		Sequencing	CTTGTACACACCGCC			

*: The primer was labeled at the 5-end with biotin.

### Protocols of pyrosequencing

Pyrosequencing was performed using PyroMark 96MA instrument (Qiagen, formerly Biotage AB, Inc.). PSQ 96MA system had two modes to detect gene mutation. One was dedicated sequence analysis (SQA) Software which could give true sequence information about 50 bases. The other was single-nucleotide polymorphism (SNP) Software which could support fast genotyping results.

To determine the optimal mutation detection modes for *rpoB*, *katG, rpsl, embB, gyrA* and *rrs* genes, 10 collected *M.tuberculosis* clinical isolates with known sequence by Sanger Sequencing were detected gene mutations by pyrosequencing with both SQA mode and SNP mode. Then the results from pyrosequencing were compared with the gold standard “Sanger sequencing” results by the Identifire software. The pyrosequencing results were considered valid if the pyrosequencing results were consistent with Sanger Sequencing results. The detection mode and the nucleotide dispensation order of pyrosequencing were considered optimum if pyrosequencing results were valid and the assay could consume less detection time and reagent. The conditions were presented in Table 2.

**Table 2:** **Optimum conditions for detection mutation of** ***rpoB*,** ***katG, gyrA, rrs, embB*** **and** ***rpsl*** **genes by pyrosequencing**

**Loci**	**Detection mode**	**Nucleotide dispensation order**
*rpoB*	SQA mode	RRDR mutation: 16(AGCT)
*katG*	SNP mode	315 mutation: GTCACAGTCTG
*rpsl*	SNP mode	43 mutation: CGATACGAGT 88 mutation: CGATGCACGC
*embB*	SNP mode	306 mutation:TGCAGTGACGCGCAGT
*gyrA*	SQA mode	QRDR mutation: 12(GACT)
*rrs*	SNP mode	1401 mutation: TCGTCAGTCAGTCAT

The gene mutations of 205 *M.tuberculosis* clinical isolates and 24 sputum samples were detected by pyrosequencing under the optimal conditions as considered above.

In brief, pyrosequencing method included preparation of the template and sequencing reaction. Firstly the PCR amplification product immobilized on the streptavidin-coated sepharose beads (GE Healthcare, Uppsala, Sweden) was converted into single-stranded DNA template and purified with a vacuum preparation tool. Then a sequencing primer was subsequently annealed to the template. Pyrosequencing was performed in an automated PyroMark 96MA instrument according to the manufacturer’s instructions. For the SQA mode detection, a PyroMark Gold Q96 SQA reagents (Qiagen, 972812) and cyclic dispensation of the nucleotide was used. The resultant sequences were compared with a database containing sequences of known mutations by mean of the Identifire software. The mutation types and loci were determined using alignment with the sequence results of *M.tuberculosis* H37Rv (ATCC25618, and GenBank accession no. NC_000962) [27]. For the SNP mode, the genotyping results were directly obtained by using the PyroMark Gold Q96 reagents (Qiagen, 972804). During pyrosequencing testing, amplified H37Rv genomic DNA was employed as a control. Some new nucleotide sequences were submitted to the Genbank (Accession numbers: KJ659894, KJ659895, KJ659896, KJ659897, KJ659898, KJ659899, KJ659900, KJ659901, KJ659902).

### DST of *M.tuberculosis* by the Bactec MGIT 960 system

DST was performed using the Bactec MGIT 960 mycobacterial detection system. Drug concentrations used in the MGIT 960 system were: RIF: 1.0 μg/ml, INH: 0.1 μg/ml, SM: 1.0 μg/ml, EMB: 5.0 μg/ml, OFL: 2.0 μg/ml and AMK: 1.0 μg/ml [28].

### Statistical analysis

Data analysis was carried out using Stata version 9 (Statacorp, Texas, USA). The sensitivity, specificity and accuracy were calculated for pyrosequencing results versus Bactec MGIT 960 system results. The Bactec MGIT 960 system results were considered as the gold standard of DST for comparison with the results obtained from pyrosequencing. The agreement between the pyrosequencing results and Bactec MGIT 960 results was analyzed using Kappa value.

## Results

### DST using Bactec MGIT 960 system

DST of RIF, INH, SM, EMB, OFL and AMK was detected by Bactec MGIT 960 system. Of the 229 re-treatment PTB patients samples, 89.5% cases were identified resistant to at least one of the tested drugs, above 50% cases were resistant to four or five tested drugs and 6.6% cases were resistant to only one drug.

### Pyrosequencing results for the detection of gene mutations

All of the 205 clinical isolates and 24 sputum samples, pyrosequencing identified 10 kinds of codon involving in 39 types of mutations in RRDP of the *rpoB* gene, and 60.4% (110/182) and 20.3% (37/182) of the RIF-resistant clinical samples had a mutation at the codon 531 and 526, respectively, in Table 3. The 74.2% (141/190) of the INH-resistant clinical samples had a mutation at codon 315 in the *katG* gene. No mutation in *katG* S315 was found in INH-susceptible clinical samples. The 65.4% (100/153) and 11.8% (18/153) of the SM-resistant clinical samples had a mutation at the codon 43 and 88, respectively, in the *rpsl* gene. The 55.5% (76/137) of the EMB-resistant clinical samples had a mutation at codon 306 in the *embB* gene. There were four kinds of codons including 12 types of mutations in the regions of the quinolone resistance-determining regions (QRDR) of the *gyrA* gene, and 62% (88/142) of the OFL-resistant clinical samples had a mutation at codon 94. The 77.3% (34/44) of the AMK-resistant clinical samples showed a mutation in position 1401 in the *rrs* gene, and no mutation in this position was found in AMK-susceptible clinical samples. Mutations of the *katG*, *embB*, *rrs*, *gyrA* and *rpsl* genes were identified by pyrosequencing shown in Table 4. The detailed information of sequences were provided in the Additional file 1

**Table 3:** **Mutations of the** ***rpoB*** **gene identified by pyrosequencing in clinical isolates and sputum samples**

**Amino acid change(s)**	**Codon change(s)**	**BACTEC MGIT 960 tests**	
		**R**	**S**
L511P	CTG/CCG	1	1
Q513H	CAA/CAC	1	
D516N	GAC/AAC	1	
D516A	GAC/GCC	1	
D516G	GAC/GGC	3	
D516V	GAC/GTC	5	
D516Y	GAC/TAC	1	1
D516F	GAC/TTC	1	
N518S	AAC/AGC	1	
H526N	CAC/AAC	4	
H526Q	CAC/CAG	1	
H526R	CAC/CGC	2	
H526D	CAC/GAC	9	
H526Y	CAC/TAC	6	
H526C	CAC/TGC	5	
H526F	CAC/TTC	1	
S531T	TCG/ACG	2	
S531Y	TCG/TAT	2	
S531W	TCG/TGG	5	
S531L	TCG/TTG	92	
L533P	CTG/CCG	1	
L533R	CTG/CGG	4	
Q510H + H526Y	CAG/CAC + CAC/TAC	1	
L511P + D516G	CTG/CCG + GAC/GGC	1	
L511P + Q513P	CTG/CCG + CAA/CCA	1	
L511P + D516G + N518S	CTG/CCG + GAC/GGC + AAC/GAC	1	
N518S + H526P	AAC/AGC + CAC/CCC	1	
S512T + D516G	AGC/ACG + GAC/GGC	1	
D516A + L533P	GAC/GCC + CTG/CCG	1	
H526N + L533P	CAC/AAC + CTG/CCG	1	
H526Q + L533P	CAC/CAG + CTG/CCG	1	
H526Y + S531T + L530R	CAC/TAC + TCG/ACG + CTG/CGT	1	
L511P + S531L	CTG/CCG + TCG/TTG	1	
D516A + S531L	GAC/GCC + TCG/TTG	1	
D516V + S531L	GAC/GTC + TCG/TTG	1	
S531L + L533R	TCG/TTG + CTG/CGG	1	
H526D + L533R	CAC/GAC + CTG/CGG	1	
H526Y + L533R	CAC/TAC + CTG/CGG	1	
S531L + L533S	TCG/TTG + CTG/TCG	2	
WT		12	45
Fail		3	

R: resistance; S: susceptibility; WT: wild type.

**Table 4:** **Mutations of the** ***katG, embB, rrs, gyrA*** **and** ***rpsl*** **genes identified by pyrosequencing in clinical isolates and sputum samples**

**Drugs**	**Genes**	**Amino acid change(s)**	**Codon change(s)**	**Nucleotide change**	**BACTEC 960 tests**	
					**R**	**S**
INH	*katG*					
		S315T	AGC/ACC		133	
		S315N	AGC/AAC		5	
		S315T	AGC/ACA		3	
		WT			46	39
		Fail			3	
SM	*rpsl*					
		K43R	AAG/AGG		100	2
		K88R	AAG/AGG		18	1
		WT			32	71
		Fail			3	2
EMB	*embB*					
		M306I	ATG/ATA		17	4
		M306I	ATG/ATC		2	2
		M306I	ATG/CTG		7	2
		M306V	ATG/GTG		50	4
		WT			57	80
		Fail			4	
OFL	*gyrA*					
		G88A	GGC/GCC		1	
		G88C	GGC/TGC		2	
		A90V	GCG/GTG		22	
		S91P	TCG/CCG		8	1
		D94N	GAC/AAC		5	
		D94H	GAC/CAC		3	
		D94A	GAC/GCC		18	
		D94G	GAC/GGC		54	
		D94Y	GAC/TAC		5	
		A90V + D94N	GCG/GTG + GAC/AAC		1	
		A90V + D94G	GCG/GTG + GAC/GGC		1	
		A90V + D94Y	GCG/GTG + GAC/TAC		1	
		WT			19	86
		Fail			2	
AMK	*rrs*					
				A1401G	34	
				WT	10	178
		Fail				7

### The performance of pyrosequencing for the detection of TB resistance

Compared with Bactec MGIT 960 method, pyrosequencing showed higher sensitivity and specificity in RIF, OFL, AMK and SM than in INH and EMB. The detection of EMB resistance showed the lowest sensitivity among six drugs not only in clinical isolates (58.7%) but also in sputum samples (41.7%). The INH and AMK showed the highest specificity (100%) in all the detecting drugs and samples. These results were present in Table 5.

**Table 5:** Comparison of pyrosequencing and Bactec MGIT 960 DST

**Drugs and specimen**	**Pyro. methods**	**No. of samples by Bactec 960 method**		**Mean% (95% CI)**			
**Clinical isolates**		**R**	**S**	**Sensitivity**	**Specificity**	**Accuracy**	**Kappa**
RIF	R	152	1	94.4	97.6	95.0	0.86
	S	9	40	89.7-97.0	87.4-99.6		
INH	R	128	0	75.3	100	79.2	0.49
	S	42	32	68.3-81.2	89.3-100.0		
SM	R	107	3	79.3	95.4	84.5	0.68
	S	28	62	71.7-85.2	87.3-98.4		
EMB	R	71	10	58.7	87.7	70.3	0.43
	S	50	71	49.8-67.1	78.7-93.2		
OFL	R	108	1	86.4	98.7	91.1	0.82
	S	17	77	79.3-91.3	93.1-99.8		
AMK	R	32	0	82.1	100	96.5	0.88
	S	7	159	67.3-91.0	97.6-100.0		
Sputum samples							
RIF	R	15	1	83.3	83.3	83.3	0.6
	S	3	5	60.8-94.2	43.7-97.0		
INH	R	13	0	76.5	100	83.3	0.66
	S	4	7	52.7-90.4	64.6-100.0		
SM	R	11	0	73.3	100	83.3	0.67
	S	4	9	48.1-89.1	70.0-100.0		
EMB	R	5	2	41.7	81.8	60.9	0.23
	S	7	9	19.3-68.1	52.3-94.9		
OFL	R	13	0	86.7	100	91.7	0.83
	S	2	9	62.1-96.3	70.1-100.0		
AMK	R	2	0	40	100	87.5	0.51
	S	3	19	11.8-76.9	83.2-100.0		

CI: confidence interval.

## Discussion

In this study, we developed a method for detecting RIF, INH, EMB, SM, OFL and AMK resistance by the pyrosequencing technology and evaluated this method on a panel of 229 clinical samples of *M.tuberculosis*. We optimized the pyrosequencing conditions to detect drug resistance to six anti-TB drugs. In the pyrosequencing operating system,two modes, SQA and SNP, could be used, but the two modes differed in several aspects. For example, SQA mode detected a short and medium length DNA fragment, and SNP mode detected only a single base variation. In our experiments, although SQA mode could detect all 6 drugs resistance, we chose the SNP mode rather than SQA mode to detect the mutation of *katG, rpsl, embB* and *rrs* based on the below reasons. The mutations of these genes were mainly involved a few point mutations. In detection resistance of INH, only *katG* gene at codon 315 was detected; in EMB, only *embB* gene at codon 306 was detected; in SM, only *rpsl* gene at codon 44 and 88 was detected; in AMK, only *rrs* gene at nucleotide position 1401 was detected. However, for *rpoB* and *gyrA* gene, we chose the SQA mode to detect the mutation because the sequence of interesting had been highly polymorphic and a short region should be sequenced. The most important advantage for choosing the SNP mode rather than SQA mode was that the former was quicker than the latter. By SNP mode, it was possible to sequence 96 samples in approximately 15 min, while it needed 70 min by the SQA mode. Thus, the time needed by SNP mode was shorter approximately only one- fourth of the time needed by SQA mode. Another advantage with SNP mode over SQA mode was that it could greatly reduce the detection cost to be 1/3.

The traditional gold standard for detection drug resistance of tuberculosis was the bacteriological methods. Therefore, we compared the results of pyrosequencing with the Bactec MGIT 960 mycobacterial detection system. The pyrosequencing approach showed different sensitivity and specificity among six drugs. For clinical isolates, the sensitivity of pyrosequencing for detecting resistance to the six drugs ranged from 58.7% to 94.4%, and the specificity was above 87.7%.

In RIF assay, compared to Bactec MGIT 960 DST, the sensitivity and specificity of the pyrosequencing method were similar to previously reported values [29]. The 95.0% (192/202) of pyrosequencing results were concordant with the results of Bactec MGIT 960. Among the ten inconsistent specimens, one isolate which was RIF susceptible was resistant by prosequecing and the mutation was detected at codon Leu 511 Pro. This mutation usually occurred in RIF resistant isolates together with other mutations [29]; therefore this mutation alone maybe had low predictive value for RIF resistance. The other nine phenotypical RIF resistant isolates did not detect a mutation in the RRDR and it was possible that another mutations existed outside the RRDR or elsewhere in the genome.

In INH assay, the specificity was high to be 100%, whereas the sensitivity of detecting resistance to INH was much low. The relatively low sensitivity in the detection of INH resistance might be related to only *katG* 315 detected. Both *inhA* and *aphC* had also been demonstrated to confer the INH resistance [14,30]. So these genes might increase the sensitivity when evaluated.

In SM assay, the codon 43 and 88 of *rpsl* gene were detected by pyrosequencing and showed low sensitivity. In previous report, the mutations in position 513 or 516 of *rrs* gene were correctly identified in SM-resistant clinical isolates [31]. Therefore, the sensitivity might increase when these genes involved.

The sensitivity and specificity of detection of EMB resistance were the lowest among six drugs, which were consistent with the previous study [32]. These results also suggested that codon 306 point mutation in the *embB* gene was a poor indicator of EMB resistance, and other genes, such as *embC* and *embA* might confer EMB resistance [33].

The high specificity of the OFL assay (98.7%) and the low sensitivity (86.4%) were analyzed here. One OFL-susceptible isolates was identified as resistant by the pyrosequencing assay because the MIC (0.5 μg/ml) for this isolate was close to the critical concentration for OFL (1.0 μg/ml). The other 17 of the 125 phenotypical OFL-resistant isolates did not detect a mutation in the QRDR of the *gyrA* gene, which might have other mutation existed other site in *gyrA* or in *gyrB* gene [34].

The specificity of AMK resistance was 100%, and the sensitivity was low (82.1%). The 7 of the 39 AMK-resistant isolates without a mutation at position 1401 in the *rrs* gene suggested that another mutation might exist in other site of *rrs* gene or other genes conferring AMK resistance. These results might provide the cues to further studies on AMK resistance.

In addition, we detected the resistance of 24 smear-positive sputum samples by pyrosequencing in re-treatment PTB patients, which showed that pyrosequencing approach could applied to detect smear-positive sputum specimens. Then the bacterial culture avoided by this direct examination of sputum samples, and the detecting turn-around time could greatly shorten. However, most of the samples in our study were quantified as 3+ to 4+ for acid-fast (AFB) staining, so this application could be used to study in large scale and in sputum specimen of 1+ or 2+ acid-fast (AFB) staining. The nested PCR might be essential when detected the sputum specimens with 1+ or 2 + .

## Conclusions

We concluded that the pyrosequencing is a rapid and high-throughput method for the detection of resistance to RIF, INH, SM, EMB, OFL and AMK in *M.tuberculosis*. Pyrosequencing can be used as a practical molecular diagnostic tool for screening and predicting the resistance for re-treatment pulmonary tuberculosis patients.

## Competing interests

The authors declare that they have no competing interests.

## Authors' contributions

RJZ, CTZ, ZYH and BXG designed the study. RJZ, QG, LHQ, JML, CTZ, HYC and ZHL performed the experiments. CTZ, RJZ and JML performed the statistical analysis. CTZ and RJZ wrote the manuscript. All authors contributed to the study and had read and approved the final manuscript. BXG is the guarantor.

## Pre-publication history

The pre-publication history for this paper can be accessed here:

http://www.biomedcentral.com/1471-2334/14/200/prepub

## Supplementary Material

Additional file 1 The detailed information of sequences in this study. Click here for file
